# GSK3 and p53 - is there a link in Alzheimer's disease?

**DOI:** 10.1186/1750-1326-5-7

**Published:** 2010-01-26

**Authors:** Carole J Proctor, Douglas A Gray

**Affiliations:** 1 Centre for Integrated Systems Biology of Ageing and Nutrition, Institute for Ageing and Health, Newcastle University, Newcastle upon Tyne, NE4 5PL, UK; 2 Ottawa Health Research Institute, Ottawa, ON K1H 8L6, Canada; 3 Department of Biochemistry, Microbiology and Immunology, University of Ottawa, Ottawa, ON K1H 8M5, Canada

## Abstract

**Background:**

Recent evidence suggests that glycogen synthase kinase-3β (GSK3β) is implicated in both sporadic and familial forms of Alzheimer's disease. The transcription factor, p53 also plays a role and has been linked to an increase in tau hyperphosphorylation although the effect is indirect. There is also evidence that GSK3β and p53 interact and that the activity of both proteins is increased as a result of this interaction. Under normal cellular conditions, p53 is kept at low levels by Mdm2 but when cells are stressed, p53 is stabilised and may then interact with GSK3β. We propose that this interaction has an important contribution to cellular outcomes and to test this hypothesis we developed a stochastic simulation model.

**Results:**

The model predicts that high levels of DNA damage leads to increased activity of p53 and GSK3β and low levels of aggregation but if DNA damage is repaired, the aggregates are eventually cleared. The model also shows that over long periods of time, aggregates may start to form due to stochastic events leading to increased levels of ROS and damaged DNA. This is followed by increased activity of p53 and GSK3β and a vicious cycle ensues.

**Conclusions:**

Since p53 and GSK3β are both involved in the apoptotic pathway, and GSK3β overactivity leads to increased levels of plaques and tangles, our model might explain the link between protein aggregation and neuronal loss in neurodegeneration.

## Background

Glycogen synthase kinase-3 (GSK3) is a protein kinase involved in many physiological processes including cell structure, metabolism, gene expression and apoptosis. There are two forms of GSK3 - GSK3α and GSK3β both of which are ubiquitously expressed and constitutively active. Many of GSK3's substrates require pre-phosphorylation (priming) and so activity of priming kinases may limit GSK3 activity. GSK3 activity is also inhibited by insulin and Wnt signalling.

GSK3 has been implicated in both sporadic and familial forms of Alzheimer's disease (AD) and this recently led Hooper et al. to put forward the 'GSK3 hypothesis of AD' [1]. This hypothesis proposes that over activity of GSK3 accounts for tau hyper-phosphorylation, increased production of Aβ, inflammatory responses, reduction in acetylcholine synthesis, and memory impairment, all typical features of AD. GSK3 is also a key mediator of apoptosis but some studies show opposing effects on apoptosis (e.g. [2,3]). A solution to this paradox is that GSK3 has a pro-apoptotic effect on the intrinsic pathway but an anti-apoptotic effect on the extrinsic pathway [4].

The tumour suppressor protein, p53, is well known for its role in cell cycle arrest, DNA repair and apoptosis and is the most frequently mutated gene in human cancer. It is also plays an important role in ageing [5]. A role for p53 in neurodegeneration is less well known but several studies have reported an increase in p53 immunoreactivity in sporadic AD (cited in Hooper et al) [6] especially in subpopulations of cortical neurons undergoing neurofibrillary degeneration. It has recently been demonstrated that expression of p53 is partly mediated by the intracellular, transcriptionally active domain of the Amyloid Precursor Protein (APP) [7] and also that Aβ, especially Aβ42 binds to the p53 promoter and enhances transcription [8]. An interesting observation by Hooper et al (2007) is that p53 induces tau phosphorylation but that the effects are indirect since they observed p53 in the nucleus and tau in the cytoskeletal compartment [6]. Such effects could either be due to a p53 target gene or a kinase which is downstream in a p53 signalling pathway. One possibility is that GSK3β is the kinase responsible for the p53-induced tau phosphorylation, since p53 affects GSK3β activity. This paper focuses on this possible link which is described further below.

AD is characterised by the presence of extracellular amyloid plaques and intracellular tangles of which tau is the principal component. There is controversy over whether these plaques and tangles are toxic to cells or whether they are in fact a protective mechanism. The main consensus is that the intermediate soluble oligomers are the most harmful as these interfere with the cellular machinery. In particular they may bind to proteasomes and inhibit proteasomal function. Proteasomes are required for the turnover of regulatory short-lived proteins such as p53 and so any decline in proteasomal function may have severe consequences for cellular homeostasis. Oxidised and damaged/misfolded proteins are also degraded by proteasomes, so less efficiency of this system may result in aggregation of other misfolded proteins, apart from Aβ and tau. Aggregated protein inhibits proteasomal function with small soluble oligomers being the most toxic. For example, Tseng et al (2008) [9] show that small oligomers but not monomers of Aβ inhibit proteasomes. Similarly, it has been shown that paired helical filaments of tau inhibit the proteasome [10].

Tau protein binds and stabilises microtubules (MT) but it also has to disassociate to allow trafficking of other molecules along MTs and so the interaction is dynamic. A widely held view is that phosphorylation of tau induces aggregation since tau tangles often contain hyperphosphorylated tau. However, it has been shown that phosphorylation does not directly increase the propensity of tau to form aggregates [11,12] but rather that the effect is due to increased pools of unbound tau since phosphorylated tau is unable to bind to microtubules. Tau is phosphorylated by several different kinases but the Cdk5 and GSK3β kinases phosphorylate the sites which are implicated in AD [13]. Normal tau is also turned over by the 20S proteasome with a half-life of about 12-14 hours [14], whereas phosphorylated tau may be ubiquitinated and degraded by the 26S proteasome.

### Link between GSK3 and p53

It has been shown that apoptotic stimuli induce nuclear accumulation of GSK3β colocalising it with p53 [15]. This study reported that there was no nuclear accumulation of GSK3β after DNA damage (induced by camptothecin treatment) but rather an exclusive activation of the nuclear pool with no activation of cytosolic pools. The authors showed that p53 coimmunoprecipitates with GSK3β from nuclear fractions after camptothecin treatment. They found that binding of p53 directly increases activity of GSK3β, and that the activated GSK3β contributes to transcriptional activity of p53. Another study showed that GSK3 can regulate p53 levels through the phosphorylation of Mdm2 [16]. GSK3 phosphorylates sites in the central domain of Mdm2 and this phosphorylation is required for p53 degradation. Inhibition of GSK3 leads to an increase in p53 levels. However p53 still binds to and is ubiquitinated by Mdm2 after GSK3 inhibition suggesting that there is a post-ubiquitination role of Mdm2 for p53 degradation.

Both GSK3 and p53 are degraded by the ubiquitin-proteasome system. GSK3β is a relatively stable protein with a half-life of about 48 hours [17], whereas p53 is a relatively unstable protein with a half-life of 20-30 minutes under normal cellular conditions. It has been shown that the function of the ubiquitin-proteasome system declines with age and so we would expect this to have an impact on protein turnover and perhaps to explain the increased level of p53 observed in the ageing brain. In particular, as mentioned earlier, the proteasome is inhibited by aggregated protein and so proteasome capacity may become overwhelmed in time.

**Figure 1:**
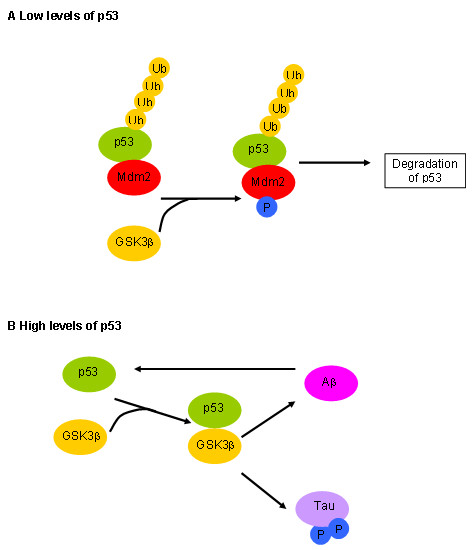
**GSK3/p53 hypothesis for AD**. A Under normal conditions p53 is bound to Mdm2 which ubiquitinates and targets p53 for proteasomal degradation. B Under conditions of stress, p53 is stabilised and it can form complex with GSK3β. This results in increased activity of GSK3β which leads to hyperphosphorylation of tau and increased production of Aβ. Aβ affects p53 pools by increasing its transcription rate and through inhibition of the proteasome. Note that we also assume that GSK3β can also bind to phosphorylated p53.

**The GSK3/p53 Hypothesis of AD **(an extension of the GSK Hypothesis)As previously discussed, under normal conditions, GSK3β is involved in the regulation of p53 by phosphorylating Mdm2 which allows p53 proteasomal degradation to proceed and hence low basal levels of p53 are maintained. However, under conditions of cellular stress, p53-Mdm2 complexes are disrupted and p53 is stabilised. During ageing, proteasome function also declines partly as a result of the accumulation of aggregated protein, resulting in increased levels of p53. This provides a sufficient pool of p53 for binding to GSK3β leading to increased activity of GSK3β. This in turn leads to an increase p53 activity leading to a positive feedback loop. The increase in GSK3β activity also leads to hyper-phosphorylation of tau and increased production of amyloid. This will lead to further inhibition of proteasome function and an ever increasing spiral ensues. The expected outcome would be apoptosis via the intrinsic pathway. In this scenario, the initiating event is either a DNA damage response or a decline in proteasome function. This is a very complex system and so we have summarised the key components in Figure 1.

## Methods

To build a quantitative model of the system it is necessary to specify all the molecular components of the system and the interactions between the different components. A mathematical model does not need to include every component and reaction in a system as this would make the model too complex to aid understanding. However, the model needs to contain enough detail to make it biologically realistic. It is generally best to start by keeping the model as simple as possible and then to add more detail if required. Using the Systems Biology Markup Language (SBML) to build the model is ideal for this approach [18]. SBML is a computer-readable format for representing biochemical reaction networks and complies to modelling standards allowing models to be portable and shared by the scientific community. There are many open source tools available for building and simulating SBML models. We have developed our own system at Newcastle University, known as BASIS (Biology of Ageing e-Science Integration and Simulation system) [19,20]. We used SBML shorthand to code the model and then converted it to full SBML using a Python script [21]. The model is publicly available on the BASIS website [22] and at the Biomodels database [23,24]. (Biomodel ID: MODEL0910130002). The SBML code is also available as supplementary material (See additional file 1: SBML code for GSK3-p53 model with IR event).

Most computer simulation models of biological systems use a deterministic approach which involves solving a set of differential equations to find the steady state of the system. However, this approach is not suitable for this model, since we are interested in the variability of cellular outcomes. For example, the time at which aggregates start to form in cells will be very variable, and the destination of aggregates will be affected by random processes. Therefore, we use stochastic simulation based on the Gillespie algorithm [25]. This means that the output of each simulation run is different for the same set of initial conditions and parameter values, unlike a deterministic simulation which always produces the same results.

**Figure 2:**
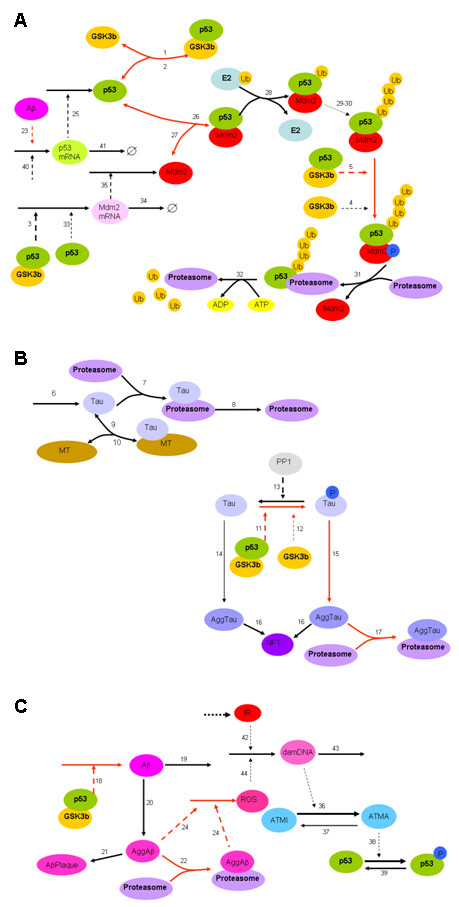
**Network diagram of the model**. A Reactions involved in p53 and Mdm2 turnover, and the interaction of p53 with GSK3β. Not all reactions involving Mdm2 degradation are included as these are similar to the reactions for p53. B Reactions involved in tau turnover and aggregation. C Reactions involved in Aβ production and aggregation, and the DNA damage response. Important reactions for this model are shown in red. Dashed lines from a species indicate that the species is a modifier of the reaction. Reaction numbers correspond to the numbers in the first column of Tables 2 and 3.

The model of the p53/Mdm2/ATM circuit (Proctor & Gray 2008) [26] was extended to include details of GSK3β, Aβ and tau. The earlier model [26] was developed to explain the oscillatory behaviour in MCF7 cells with data supplied by Uri Alon [27]. It contained details of p53 and Mdm2 turnover but the reactions for protein degradation were simplified and did not include details of ubiquitination. Since GSK3β is involved in p53 degradation via phosphorylation of Mdm2 which is in complex with polyubiquitinated p53, it is necessary to add these steps. A model of the ubiquitin-proteasome has already been developed (Proctor et al 2007) [28] and so parts of this model were incorporated into the p53 model. Figure 2 shows a diagram of the overall reaction system with the important parts of the network shown in red. Table 1 lists all the species in the model, Table 2 shows all the reactions concerning GSK3β, Aβ and tau and Table 3 shows the reactions involved in p53 and Mdm2 turnover. The previous p53/Mdm2 model included a module for DNA damage to mimic the experimental system of irradiating cells and the emergence of oscillations in p53 and Mdm2 after damage. We considered two mechanisms which led to these oscillations: sequestering of Mdm2 from the p53/Mdm2 complex by the oncoprotein ARF; or activation of ATM which then phosphorylated both p53 and Mdm2 and so inhibits their binding. Either mechanism leads to an increase in levels of p53 which is followed by an increase in Mdm2 levels. As Mdm2 levels rose, complexes of p53/Mdm2 are able to reform leading to an increase in p53 degradation and so levels decrease again (see Proctor & Gray [26] for full details of modelling the oscillatory behaviour of p53, and Lahav et al [29] for the experimental details). This negative feedback loop leads to oscillations, which persist as long as DNA damage remains, keeping cells in an arrested state while DNA is repaired. The oscillations also prevent p53 levels reaching levels which would trigger apoptosis.

**Table 1:** List of model species

Name	Description	Database term	Initial amount
GSK3b	Unbound GSK3β protein	P49841	500

GSK3b_p53	GSK3β bound to p53	P49841, P04637	0

GSK3b_p53_P	GSK3β bound to phosphorylated p53	P49841, P04637	0

p53	Unbound p53 protein	P04637	5

Mdm2	Unbound Mdm2 protein	Q00987	5

Mdm2_p53	Mdm2/p53 complex	Q00987, P04637	95

Mdm2_mRNA	Mdm2 messenger RNA	SBO:0000278	10

p53_mRNA	p53 messenger RNA	SBO:0000278	10

p53_P	Phosphorylated p53	P04637	0

Mdm2_P	Phosphorylated Mdm2	Q00987	0

Ub	Ubiquitin	P62988	4000

E1	Ubiquitin activating enzyme	IPR000011	100

E2	Ubiquitin conjugating enzyme	IPR000608	100

E1_Ub	E1 bound by Ub	IPR000011, P62988	0

E2_Ub	E2 bound by Ub	IPR000608, P62988	0

p53DUB	Deubiquitinating enzyme for p53	IPR001394	200

Mdm2DUB	Deubiquitinating enzyme for Mdm2	IPR001394	200

Mdm2_p53_Ub	Monoubiquitinated p53	Q00987, P04637, P62988	0

Mdm2_p53_UbX (X = 2-4)	Polyubiquitinated p53	Q00987, P04637, P62988	0

Mdm2_P1_p53_Ub4	Phosphorylated Mdm2 bound to p53	Q00987, P04637, P62988	0

Mdm2_Ub	Monoubiquitinated Mdm2	Q00987, P62988	0

Mdm2_P_Ub	Monoubiquitinated phospho-Mdm2	Q00987, P62988	0

Mdm2_UbX (X = 2-4)	Polyubiquitinated Mdm2	Q00987, P62988	0

Mdm2_P_UbX (X = 2-4)	Polyubiquitinated phospho-Mdm2	Q00987, P62988	0

Proteasome	26S Proteasome complex	GO:0000502	500

p53_Ub4_Proteasome	p53 bound to proteasome	P04637, P62988, GO:0000502	0

Mdm2_Ub4_Proteasome	Mdm2 bound to proteasome	Q00987, P62988, GO:0000502	0

Mdm2_P_Ub4_Proteasome	phospho-Mdm2 bound to proteasome	Q00987, P62988, GO:0000502	0

ATMI	Inactive ATM	Q13315	200

ATMA	Active ATM	Q13315	0

damDNA	Amount of damaged DNA	CHEBI16991	0

IR	Dummy species to represent gamma-irradiation	-	0

ROS	Reactive oxygen species	CHEBI:26523	0

basalROS	Basal pool of ROS	CHEBI:26523	10

Abeta	Amyloid beta	P05067	0

AggAbeta	Small aggregate of Aβ	P05067	0

AbetaPlaque	Aβ plaque	P05067	0

AggAbeta_Proteasome	AggAbeta bound to the proteasome	P05067, GO:0000502	0

Tau	tau	IPR002955 IPR002955	0

Proteasome_Tau	tau bound to 20S proteasome	GO:0000502,	0

Tau_P1	tau phosphorylated by GSK3β at one site	IPR002955	0

Tau_P2	tau phosphorylated by GSK3β at two sites	IPR002955	0

MT_Tau	tau bound to the microtubule	IPR015562	100

AggTau	small aggregate of tau	IPR002955	0

AggTau_Proteasome	AggTau bound to proteasome	IPR002955, GO:0000502	

NFT	tau neurofibrillary tangle	IPR002955	0

PP1	phosphatase	P62136	50

ATP	Adenosine triphosphate	CHEBI:15422	10000

ADP	Adenosine diphosphate	CHEBI:16761	1000

AMP	Adenosine monophosphate	CHEBI:22254	1000

Terms starting with:

P or Q are from UniProtKB/Swiss-Prot [40], SBO are from Systems Biology Ontology [41], CHEBI are from Chemical Entities of Biological Interest Database [42], GO are from Gene Ontology [43], IPR are from Interpro [44]

**Table 2:** Reactions involving Gsk3β, Aβ and tau

No.	Name		Reactants and products		Kinetic Law^a^		Parameter value
1	GSK3b_p53Binding		GSK3b+p53→ GSK3b_p53		*k*_*binGSK*3*bp*53_<#GSK3b><#p53>		2.0E-6 molecule^-1 ^s^-1^

2	GSK3b_p53Release		GSK3b_p53→ GSK3b+p53		*k*_*relGSK*3*bp*53_<#GSK3b_p53>		2.0E-3 s^-1^

(1)	GSK3b_p53_PBinding		GSK3b+p53_P→ GSK3b_p53_P		*k*_*binGSK*3*bp*53_<#GSK3b><#p53_P>		2.0E-6 molecule^-1 ^s^-1^

(2)	GSK3b_p53_PRelease		GSK3b_p53_P→ GSK3b+p53_P		*k*_*relGSK*3*bp*53_<#GSK3b_p53_P>		2.0E-3 s^-1^

3	Mdm2mRNASynthesis3		GSK3b_p53→ GSK3b_p53+Mdm2mRNA		*k*_*synMdm*2*mRNAGSK*3*bp*53_<#GSK3b_p53>		7.0E-4 s^-1^

(3)	Mdm2mRNASynthesis4		GSK3b_p53_P→ GSK3b_p53_P+Mdm2mRNA		*k*_*synMdm*2*mRNAGSKbp*53_<#GSK3b_p53_P>		7.0E-4 s^-1^

4	Mdm2GSK3phosphorylation1		Mdm2_p53_Ub4+GSK3b→ Mdm2_P1_p53_Ub4+GSK3b		*k*_*phosMdm*2*GSK*3*b*_<#Mdm2_p53_Ub4> <#GSK3b>		5.0E-3 molecule^-1 ^s^-1^

5	Mdm2GSK3phosphorylation2		Mdm2_p53_Ub4+GSK3b_p53 → Mdm2_P1_p53_Ub4 +GSK3b_p53		*k*_*phosMdm*2*GSK*3*bp*53_<#Mdm2_p53_Ub4> <#GSK3b_p53>		5.0E-1 molecule^-1 ^s^-1^

(5)	Mdm2GSK3phosphorylation3		Mdm2_p53_Ub4+GSK3b_p53_P→ Mdm2_P1_p53_Ub4+GSK3b_p53_P		*k*_*phosMdm*2*GSK*3*bp*53_<#Mdm2_p53_Ub4> <#GSK3b_p53_P>		5.0E-1 molecule^-1 ^s^-1^

6	TauSynthesis		Source→Tau		*k*_*synTau*_		8.0E-5 molecule s^-1^

7	TauProteasomeBinding		Tau+Proteasome→Proteasome_Tau		*k*_*binTauProt*_*<#*Tau><#Proteasome>		1.9E-7 molecule^-1 ^s^-1^

8	TauDegradation		Proteasome_Tau→Proteasome		*k*_*degTau*_<#Proteasome_Tau>		1.0E-2 s^-1^

9	TauMTbinding		Tau→MT_Tau		*k*_*binMTTtau*_<#Tau>		1.0E-1 s^-1^

10	TauMTrelease		MT_Tau→Tau		*k*_*relMTTau*_<#MT_Tau>		1.0E-4 s^-1^

11	Tauphosphorylation1		GSK3b_p53+Tau→GSK3b_p53+Tau_P1		*k*_*phospTauGSK*3*bp*53_<#GSK3b_p53><#Tau>		1.0E-1 molecule^-1 ^s^-1^

(11)	Tauphosphorylation2		GSK3b_p53+Tau_P1→GSK3b_p53+Tau_P2		*k*_*phospTauGSK*3*bp*53_<#GSK3b_p53> <#Tau_P1>		1.0E-1 molecule^-1 ^s^-1^

(11)	Tauphosphorylation3		GSK3b_p53_P+Tau→GSK3b_p53_P+ Tau_P1		*k*_*phospTauGSK*3*bp*53_<#GSK3b_p53_P><# Tau>		1.0E-1 molecule^-1 ^s^-1^

(11)	Tauphosphorylation4		GSK3b_p53_P + Tau_P1→GSK3b_p53_P+ Tau_P2		*k*_*phospTauGSK*3*bp*53_<# GSK3b_p53_P> <#Tau_P1>		1.0E-1 molecule^-1 ^s^-1^

12	Tauphosphorylation5		GSK3b+Tau→GSK3b+Tau_P1		*k*_*phospTauGSK*3*b*_<#GSK3b><#Tau>		2.0E-4 molecule^-1 ^s^-1^

(12)	Tauphosphorylation6		GSK3b+Tau_P1→GSK3b+Tau_P2		*k*_*phospTauGSK*3*b*_<#GSK3b><#Tau_P1>		2.0E-4 molecule^-1 ^s^-1^

(13)	Taudephosphorylation1		Tau_P2+PP1→Tau_P1+PP1		*k*_*dephospTau*_<#Tau_P2><#PP1>		1.0E-2 molecule^-1 ^s^-1^

14	TauAggregation1		2Tau→2AggTau		*k*_*aggTau*_*<# *Tau><#(Tau-1)>/2.0		1.0E-8 molecule^-1 ^s^-1^

(14)	TauAggregation2		Tau+AggTau→2AggTau		*k*_*aggTau*_<#Tau><#AggTau>		1.0E-8 molecule^-1 ^s^-1^

15	TauP1Aggregation1		2Tau_P1→2AggTau		*k*_*aggTauP*1 _<#Tau_P1><#(Tau_P1-1)>/2.0		1.0E-8 molecule^-1 ^s^-1^

(15)	TauP1Aggregation2		Tau_P1+AggTau→2AggTau		*k*_*aggTauP*1 _<# Tau_P1><#AggTau>		1.0E-8 molecule^-1 ^s^-1^

(15)	TauP2Aggregation1		2Tau_P2→2AggTau		*k*_*aggTauP*2 _<#Tau_P2><# (Tau_P2-1)>/2.0		1.0E-7 molecule^-1 ^s^-1^

(15)	TauP2Aggregation2		Tau_P2 + AggTau→2AggTau		*k*_*aggTauP*2 _<#Tau_P2><# AggTau>		1.0E-7 molecule^-1 ^s^-1^

16	TangleFormation1		2AggTau→2NFT		*k*_*tangfor*_<#AggTau><#(AggTau-1)>/2.0		1.0E-3 molecule^-1 ^s^-1^

(16)	TangleFormation2		AggTau+NFT→2NFT		*k*_*tangfor*_<#AggTau><#NFT>		1.0E-3 molecule^-1 ^s^-1^

17	ProteasomeInhibitionAggTau		AggTau+Proteasome→ AggTau_Proteasome		*k*_*inhibprot*_<#AggTau><#Proteasome>		1.0E-5 molecule^-1 ^s^-1^

18	Abetaproduction1		GSK3b_p53→Abeta+GSK3b_p53		*k*_*prodAbeta*_<#GSK3b_p53>		5.0E-5 s^-1^

(18)	Abetaproduction2		GSK3b_p53_P→Abeta +GSK3b_p53_P		*k*_*prodAbeta*_<#GSK3b_p53_P>		5.0E-5 s^-1^

19	AbetaDegradation		Abeta→Sink		*k*_*degAbeta*_<#Abeta>		1.0E-4 s^-1^

20	AbetaAggregation1		2Abeta→AggAbeta		*k*_*aggAbeta*_<#Abeta><#(Abeta-1)>/2.0		1.0E-8 molecule^-1 ^s^-1^

(20)	AbetaAggregation2		Abeta+AggAbeta→2AggAbeta		*k*_*aggAbeta*_<#Abeta><#AggAbeta>		1.0E-8 molecule^-1 ^s^-1^

21	AbetaPlaqueFormation1		2AggAbeta→2AbetaPlaque		*k*_*pf *_<#AggAbeta><#(AggAbeta-1)>/2.0		1.0E-3 molecule^-1 ^s^-1^

(21)	AbetaPlaqueFormation2		AggAbeta+AbetaPlaque→2AbetaPlaque		*k*_*pf *_<#AggAbeta><#AbetaPlaque>		1.0E-3 molecule^-1 ^s^-1^

22	ProteasomeInhibitionAbeta		AggAbeta+Proteasome→ AggAbeta_Proteasome		*k*_*inhibprot*_<#AggAbeta><#Proteasome>		1.0E-5 molecule^-1 ^s^-1^

23	p53transcriptionViaAbeta		Abeta→p53_mRNA+Abeta		*k*_*synp*53*mRNAAbeta*_<#Abeta>		1.0E-5 s^-1^

24	AbetaROSproduction1		AggAbeta→AggAbeta + ROS		*k*_*genROSAbeta*_<#AggAbeta>		1.0E-5 s^-1^

24	AbetaROSproduction2		AggAbeta_Proteasome→AggAbeta_Proteasome + ROS		*k*_*genROSAbeta*_<#AggAbeta_Proteasome>		1.0E-5 s^-1^

^a ^<#X> means number of molecules of species X

We assume that the reaction volume is equal to one.

Reactions with number in 1^st ^column are shown in Figure 2; reactions with numbers in parentheses are similar to the reactions shown in Figure 2.

**Table 3:** Reactions involved in p53 turnover and the DNA damage response

No.	Name		Reactants and products		**Kinetic law**^a^		Parameter values
25	p53 synthesis		p53_mRNA→p53+p53_mRNA		*k*_*synp*53 _<#p53_mRNA>		7.0E-3 s^-1^

26	p53 Mdm2 binding		p53+Mdm2 → Mdm2_p53		*k*_*binMdm*2*p*53_<#p53><#Mdm2>		3.0E-3 molecule^-1 ^s^-1^

27	Mdm2_p53 release		Mdm2_p53→p53+Mdm2		*k*_*relMdm*2*p*53 _<#Mdm2_p53>		3.0E-5 s^-1^

	E1/Ub binding		E1+Ub+ATP→E1_Ub+AMP		*k*_*binE*1*Ub *_<#E1><#Ub><#ATP>/(5000 +<#ATP>)		2.0E-4 molecule^-1 ^s^-1^

	E2/Ub binding		E1_Ub+E2→E2_Ub+E1		*k*_*binE*2*Ub*_<#E2><#E1_Ub>		1.0E-3 molecule^-1 ^s^-1^

28	p53 ubiquitination		Mdm2_p53+E2_Ub→Mdm2_p53_Ub+E2		*k*_*p*53*Ub*_<#Mdm2_p53><#E2_Ub>		5.0E-5 molecule^-1 ^s^-1^

29	p53 polyubiquitination1		Mdm2_p53_Ub+E2_Ub→Mdm2_p53_ Ub2+E2		*k*_*p*53*PolyUb*_<#Mdm2_p53_Ub><#E2_Ub>		1.0E-2 molecule^-1 ^s^-1^

30	p53 polyubiquitinationX (X = 2-3)		Mdm2_p53_Ub(X)+E2_Ub→ Mdm2_p53_Ub(X+1)+E2		*k*_*p*53*PolyUb*_<#Mdm2_p53_Ub(X)><#E2_Ub>		1.0E-2 molecule^-1 ^s^-1^

	p53 de-ubiquitination1		Mdm2_p53_Ub+p53DUB → Mdm2_p53+p53DUB+Ub		*k*_*actDUBp*53_<#Mdm2_p53_Ub> <#p53DUB>		1.0E-7 molecule^-1 ^s^-1^

	p53 de-ubiquitinationX (X = 2-4)		Mdm2_p53_Ub(X)+p53DUB → Mdm2_p53_Ub(X-1)+p53DUB+Ub		*k*_*actDUBp*53_<#Mdm2_p53_Ub(X)> <#p53DUB>		1.0E-7 molecule^-1 ^s^-1^

31	p53 Proteasome binding		Mdm2_P_p53_Ub4+Proteasome→ p53_Ub4_Proteasome+Mdm2		*k*_*binProt*_*<# *Mdm2_P_p53_Ub4><#Proteasome>		2.0E-6 molecule^-1 ^s^-1^

32	p53 degradation		p53_Ub4_Proteasome+ATP→ 4Ub+Proteasome+ADP		*k*_*degp*53_<#p53_Ub4_Proteasome> <#ATP>/(5000 + <#ATP>)		1.0E-2 s^-1^

33	Mdm2mRNA synthesis1		p53→p53+Mdm2_mRNA		*k*_*synMdm*2*mRNA*_<#p53>		5.0E-4 s^-1^

(33)	Mdm2mRNA synthesis2		p53_P→p53_P+Mdm2_mRNA		*k*_*synMdm*2*mRNA*_<#p53_P>		5.0E-4 s^-1^

34	Mdm2 mRNA degradation		Mdm2_mRNA→Sink		*k*_*degMdm*2*mRNA*_<#Mdm2_mRNA>		5.0E-4 s^-1^

35	Mdm2 synthesis		Mdm2_mRNA→Mdm2_mRNA+Mdm2		*k*_*synMdm*2_<#Mdm2_mRNA>		4.95E-4 s^-1^

	Mdm2 ubiquitination^b^		Mdm2+E2_Ub→Mdm2_Ub+E2		*k*_*Mdm*2*Ub*_<#Mdm2><#E2_Ub>		4.56E-6 molecule^-1 ^s^-1^

	Mdm2 polyubiquitination1^c^		Mdm2_Ub+E2_Ub→Mdm2_Ub2+E2		*k*_*Mdm*2*PolyUb*_<#Mdm2_Ub><#E2_Ub>		4.56E-3 molecule^-1 ^s^-1^

	Mdm2 polyubiquitinationX^c^ (X = 2-3)		Mdm2_Ub(X)+E2_Ub→Mdm2_Ub(X+1)+E2		*k*_*Mdm*2*PolyUb*_<#Mdm2_Ub(X)><#E2_Ub>		4.56E-3 molecule^-1 ^s^-1^

	Mdm2 de-ubiquitination1^c^		Mdm2_Ub+Mdm2DUB → Mdm2+Mdm2DUB+Ub		*k*_*actDUBMdm*2_<#Mdm2_Ub><#Mdm2DUB>		1.0E-7 molecule^-1 ^s^-1^

	Mdm2 de-ubiquitinationX^c^ (X = 2-4)		Mdm2_Ub(X)+Mdm2DUB → Mdm2_Ub(X-1)+Mdm2DUB+Ub		*k*_*actDUBMdm*2_<#Mdm2_Ub(X)> <#Mdm2DUB>		1.0E-7 molecule^-1 ^s^-1^

	Mdm2 proteasome binding^c^		Mdm2_Ub4 + Proteasome→Mdm2_Ub4_Proteasome		*k*_*binProt*_*<# *Mdm2_Ub4><#Proteasome>		2.0E-6 molecule^-1 ^s^-1^

	Mdm2 degradation^c^		Mdm2_Ub4_Proteasome+ATP→ 4Ub+Proteasome+ADP		*k*_*degMdm*2_<#Mdm2_Ub4_Proteasome> <#ATP>/(5000+<#ATP>)		1.0E-2 molecule^-1 ^s^-1^

36	ATM activation		damDNA+ATMI→damDNA+ATMA		*k*_*actATM*_<#damDNA><#ATMI>		1.0E-4 molecule^-1 ^s^-1^

37	ATM inactivation		ATMA → ATMI		*k*_*inactATM*_<#ATMA>		5.0E-4 s^-1^

38	p53 phosphorylation		p53+ATMA→p53_P+ATMA		*k*_*phosp*53_<#p53><#ATMA>		2.0E-4 molecule^-1 ^s^-1^

39	p53 dephosphorylation		p53_P→p53		*k*_*dephosp*53_<#p53_P>		5.0E-1 s^-1^

	Mdm2 phosphorylation		Mdm2+ATMA→Mdm2_P+ATMA		*k*_*phosMdm*2_<#Mdm2><#ATMA>		2.0 molecule^-1 ^s^-1^

	Mdm2 de-phosphorylation		Mdm2_P→Mdm2		*k*_*dephosMdm*2_<#Mdm2_P>		5.0E-1 s^-1^

40	p53mRNA synthesis		Source→p53_mRNA		*k*_*synp*53*mRNA*_		1.0E-3 molecule s^-1^

41	p53mRNA degradation		p53_mRNA→Sink		*k*_*degp*53*mRNA*_<#p53_mRNA>		1.0E-4 s^-1^

42	DNA damage by IR		IR→damDNA+ IR		*k*_*dam*_<#IR>		8.0E-2 s^-1^

43	DNA repair		damDNA→Sink		*k*_*repair*_<#damDNA>		2.0E-5 s^-1^

44	DNA damage by ROS		ROS→damDNA+ROS		*k*_*damROS*_<#ROS>		1.0E-5 s^-1^

(44)	DNA damage by basalROS		basalROS→damDNA+ basalROS		*k*_*dambasalROS*_<#basalROS>		1.0E-9 s^-1^

^a ^<#X> means number of molecules of species X, ^b^Mdm2_P is also ubiquitinated in the same way but the first step occurs at a higher rate (*k*_*Mdm*2*PUb *_= 6.84E-6). ^c^Mdm2_P undergoes polyubiquitination, de-ubiquitination, binding to the proteasome and degradation at the same rate as Mdm2. We assume that the reaction volume is equal to one. Reactions with number in 1^st ^column are shown in Figure 2; reactions with numbers in parentheses are similar to the reactions shown in Figure 2.

GSK3β has a relatively long half-life (about 48 hours) [17] and like p53, it is turned over by the proteasome. It has been shown that Hsp90 maintains the stability of GSK3β [30]. This study also showed that Hsp90 inhibition results in proteasome-dependent degradation of GSK3β, and subsequently reduces tau phosphorylation. However, we chose not to include GSK3β turnover in this model because it would be stable under the conditions we are simulating and we aim to keep the model as simple as possible. GSK3β binds to p53 and we assume that it binds to either phosphorylated or unphosphorylated p53 but that it does not bind to p53/Mdm2 complexes. GSK3β has higher activity when bound to p53. p53 bound by GSK3β also has increased transcriptional activity, as shown by Watcharisit et al. (2003) [31] whereby inhibition of GSK3β leads to 50% reduction in Mdm2 mRNA.

We assume that GSK3β phosphorylates tau with increased activity if GSK3β is bound to p53. We also include de-phosphorylation of tau by a phosphatase. We also assume that GSK3β_p53 produces Aβ, however, under normal conditions Aβ is removed by degradation and does not accumulate. We expect that under conditions of stress, levels of GSK3β_p53 would increase and that there would also be increased Aβ production. Aβ may form small aggregates which could either inhibit proteasomes or form plaques. We assume that plaques, being outside the cell, do not interfere with proteasomes. We also assume that Aβ increases the transcription of p53. We assume that tau is normally bound to microtubules but that binding is prevented when tau is phosphorylated. We also include tau turnover in the model and assume that unphosphorylated tau is degraded by the proteasome without the need for ubiquitination.

The parameter values were taken from experimental data where possible. For example we set the degradation rates for p53, Mdm2 and tau based on their half-life of 20 minutes, 30 minutes and 12 hours respectively and then set the synthesis rates so that the proteins stayed at basal levels under unstressed conditions. We set aggregation rates to be low so that no aggregation would take place under unstressed conditions. It is known that phosphorylation reactions take place on time scales in the order of minutes so we assumed that these reactions are fast. Further details of the parameters involved in the DNA damage response are given in [26]. Where parameter values are unknown, the values were adjusted so that the system was at steady state under unstressed conditions. The model was validated by checking the model predictions for levels of p53 and Mdm2 after the irradiating event.

## Results

### Model predictions for normal conditions

**Figure 3:**
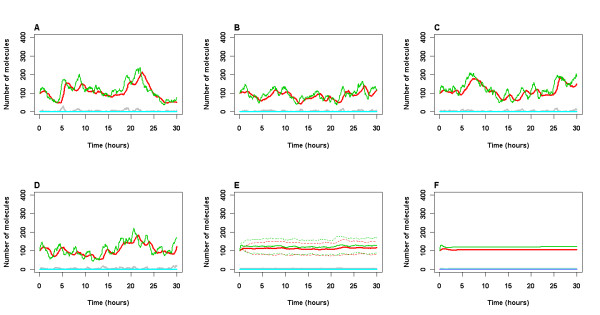
**Model output for normal (unstressed) conditions**. A-D Four individual simulations over 30 hour time period. E Mean (solid lines) and standard deviation (dashed lines) of 100 simulations. F Deterministic solution from Cell Designer. All parameter values used are given in Tables 2 and 3. Levels of p53 (total pool, including bound and ubiquitinated species), Mdm2 (total pool, including bound and ubiquitinated species), p53 bound to GSK3β, damaged DNA (damDNA), Aβ plaques and Tau tangles are shown. Key: green line: p53, red line: Mdm2, gray line: GSK3b_p53, purple line: damaged DNA, blue line: Tau tangles, cyan line: Abeta plaques.

As we used stochastic simulation, each simulation run produces different output for the same set of initial conditions. Therefore, it is necessary to carry out repeat simulations to examine the variability. The model output for 4 individual runs is shown in Figure 3A-D. When there is no cellular stress, no DNA damage occurs, p53 levels remain low and p53 is in complex with Mdm2. Therefore, only very low levels of GSK3β are able to bind to p53 and so the model predicts that there is a very low rate of either Aβ production or tau phosphorylation. Figure 3E shows the mean levels and standard deviations from 100 simulations and it can be seen that this is similar to the results of a deterministic simulation (see Figure 3F) using Cell Designer [32]. Note that the standard deviation is very low for most of the model species apart from total p53 and total Mdm2 levels. The larger variability in levels of p53 and Mdm2 is due to the fluctuations in protein levels occurring at slightly different time-points in each simulation.

### Stressed conditions

**Figure 4:**
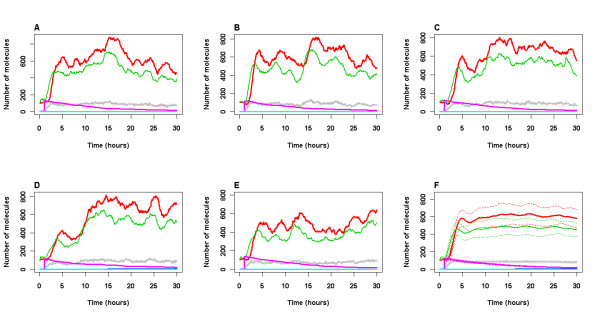
**Model output for stressed conditions**. A-E Five simulations over 30 hour time period. F Mean (solid lines) and standard deviation (dashed lines) of 100 simulations. An event to mimic irradiation took place 1 hour after the start of each simulation. All parameter values used are given in Tables 2 and 3. Levels of p53 (total pool, including bound and ubiquitinated species), Mdm2 (total pool, including bound and ubiquitinated species), p53 bound to GSK3β, damaged DNA (damDNA), Aβ plaques and Tau tangles are shown. Key: green line: p53, red line: Mdm2, gray line: GSK3b_p53, purple line: damaged DNA, blue line: Tau tangles, cyan line: Abeta plaques.

To mimic the experimental procedure of irradiating cells, we used a discrete time event to increase the value of the dummy species IR, which represents irradiation, to 25 dGy, for a period of 1 minute. This results in damaged DNA as can be seen in Figure 4. The damaged DNA activates ATM which results in phosphorylation of p53 and Mdm2 and the model predicts oscillations of both proteins as in our previous model (compare Figure 11 of Proctor & Gray, 2008[26] with Figure 4 below). Since p53 levels increase, more GSK3β is able to bind to p53 and there is a slight increase in Aβ and phosphorylated tau but no aggregation within the time scale simulated. If we simulate the model under stress conditions for longer time periods, then the oscillations die away as the damaged DNA is repaired. This is followed by clearance of Aβ and dephosphorylation of tau and so no aggregation will take place.

### Decline in repair capacity

**Figure 5:**
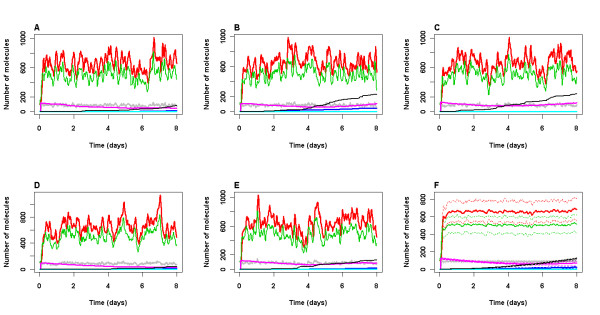
**Model output for less efficient DNA repair**. A-E Five simulations over 8 day time period. F Mean (solid lines) and standard deviation (dashed lines) of 100 simulations. An event to mimic irradiation took place 1 hour after the start of the simulation. The parameter for DNA repair was reduced by an order of magnitude (*k*_*repair *_= 2.0E-6). All other parameter values used are given in Tables 2 and 3. Levels of p53 (total pool, including bound and ubiquitinated species), Mdm2 (total pool, including bound and ubiquitinated species), p53 bound to GSK3β, damaged DNA (damDNA), ROS levels (scaled by a factor of 5 to be seen more clearly), Aβ plaques and Tau tangles are shown. Key: green line: p53, red line: Mdm2, gray line: GSK3b_p53, purple line: damaged DNA, blue line: Tau tangles, cyan line: Abeta plaques, black line: ROS levels ×5.

In order to examine whether aggregation would occur if DNA damage persisted we decreased the rate of DNA repair by an order of magnitude and simulations were run for 8 days. In this case, the model predicts that aggregates of Aβ and tau slowly start to accumulate (Figure 5). Since they are irreversible at this stage, they will persist even when DNA damage is eventually repaired. In some simulations, DNA damage starts to increase again (e.g. Figure 5B). We have also plotted ROS levels in Figure 5 and it can be clearly seen that in the simulations where DNA damage increases, there is also an increase in ROS due to the toxic effects of the aggregates.

### Increased aggregation rate

**Figure 6:**
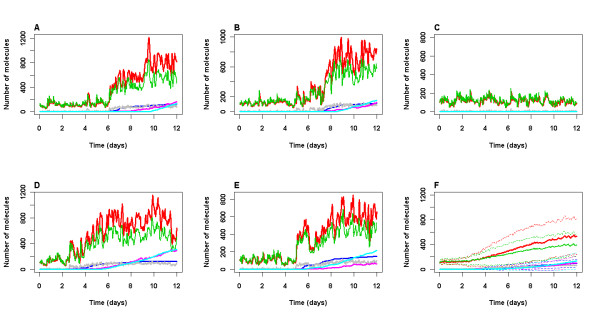
**Model output for increased aggregation rate**. A-E Five simulations over 12 day time period. F Mean (solid lines) and standard deviation (dashed lines) of 100 simulations. No irradiation. The parameters for aggregation were increased by two orders of magnitude. (*k*_*aggTauP *_= *k*_*aggTauP*1 _= *k*_*aggAbeta *_= 1.0E-6, *k*_*aggTauP*2 _= 1.0E-5). All other parameter values used are given in Tables 2 and 3. Levels of p53 (total pool, including bound and ubiquitinated species), Mdm2 (total pool, including bound and ubiquitinated species), p53 bound to GSK3β, damaged DNA (damDNA), Aβ plaques and Tau tangles are shown. Key: green line: p53, red line: Mdm2, gray line: GSK3b_p53, purple line: damaged DNA, blue line: Tau tangles, cyan line: Abeta plaques.

If the model with parameters for normal conditions was simulated for a very long period of time, eventually we would expect that either Aβ or tau might start to accumulate by chance in some cells due to stochastic effects. Many runs would be required over long periods of time to observe this and so would be very computer intensive. Therefore, to speed up the process, we increased the rate at which either Aβ or tau aggregates by two orders of magnitude and ran simulations for a period of 12 days. Figure 6 shows that there is quite a lot of cell variability with 1 cell showing no increase in aggregation but with an increase in aggregation in the other 4 simulated cells. We found that about 24% of cells do not accumulate DNA damage from the model output of 100 simulations and in these cells there is no increase in p53 levels, GSK3β activity, tau phosphorylation or an increase in Aβ. The time at which aggregates start to from is very variable with about 50% of simulated cells without any plaques by 8 days, whereas two cells start to form plaques by day 3. The model output suggests that stochastic effects in protein levels lead to an increase in levels of p53 which binds to GSK3β and so activity of both p53 and GSK3β increase. This leads to phosphorylation of tau which then starts to aggregate, and also an increase in Aβ levels. The increase in aggregation leads to increase levels of ROS and so DNA damage rates increase resulting in stabilisation of p53. Finally the activity of p53 and GSK3β increases further, leading to even more aggregation and a vicious cycle ensues. Note that the variance of p53 and Mdm2 increases with time in these simulations (Figure 6F). This is due to the levels of both proteins increasing with time in the majority of simulated cells and a small proportion of cells with low levels of these proteins throughout the simulation.

Interestingly, the model predicts that tau tangles start to form prior to Aβ plaques but that once Aβ plaques start to form they rapidly increase in size. Since aggregates can only be detected when they are a certain size, this might explain why Aβ plaques are often detected before tau tangles [33]. According to the model, the formation of tau tangles and Aβ plaques are independent events but both occur as a result of the increased activity of GSK3β. These points are discussed in more detail below.

### GSK3β and p53 null models

We also performed simulations with either GSK3β or p53 deleted from the model to examine whether the interaction between GSK3β and p53 was necessary to obtain aggregation of Aβ and tau. In order to do this it was necessary to make some slight modifications to our model. As we had assumed that phosphorylation of Mdm2 by GSK3β was necessary for p53 degradation, the model would predict that p53 levels would increase with time if there is no GSK3β. Therefore we added a reaction of p53 binding to the proteasome without Mdm2 phosphorylation but assumed that this happened at a much lower rate. The GSK3β showed similar results to the original model with regards to p53 oscillations after DNA damage (data not shown). However, if we run the model without the irradiation event and increase the aggregation rates of tau and Aβ by two orders of magnitude, then the model predicts that no aggregation takes place by 12 days indicating that GSK3β activity is necessary for tangle and plaque formation in this model (data not shown).

For the p53 null model, we added a reaction to allow Aβ formation via GSK3β even when it is not bound to p53. We assumed that this occurred at a rate *k*_*basalprodAbeta *_= 1.0E-7, (which is five hundred times lower than *k*_*prodAbeta*_, the rate corresponding to Aβ production by GSK3β which is bound to p53). We ran the model under conditions of no irradiation and high aggregation rates and found that GSK3β had to be increased 20-fold to get similar levels of plaques and tangles as seen in Figure 6 (data not shown). Therefore, although p53 is not essential for aggregation to take place, our model predicts that p53 has a substantial effect on the kinetics of aggregation. These predictions could be tested in the laboratory.

## Discussion

We extended the model of Proctor & Gray (2008) [26] to include details of p53 and Mdm2 ubiquitination, the interaction of p53 with GSK3β and the activity of GSK3β. As we had used SBML to build the models, it was straight forward to make the necessary modifications. As in our previous model, we find that a sudden increase in DNA damage leads to oscillations of p53 and Mdm2. Our new model shows that the disruption of the Mdm2/p53 complex, allows the formation of GSK3β/p53 complexes which results in increased transcriptional activity of p53 and increased kinase activity of GSK3β. The result is an increase in Aβ production, an increase in Mdm2 mRNA and an increase in tau phosphorylation. Under normal conditions, the model predicts that Aβ is cleared from cells and so does not accumulate, and tau is dephosphorylated to maintain the correct balance of phosphorylated and unphosphorylated tau. However, after a stress event, the DNA damage response leads to increased activity of p53 and GSK3β which results in increased production of Aβ and increased phosphorylation of tau. However, the parameter for DNA repair was set so that most DNA damage is repaired by 24 hours, and then Aβ is cleared and tau is dephosphorylated so that aggregates do not accumulate. In the ageing brain we would expect that DNA damage might sometimes persist either due to a decline in repair mechanisms or an increase in ROS production. We used our model to examine the outcome of a deficiency in DNA repair. In this case, we find that aggregates are much more likely to accumulate which in turn lead to increased ROS production and further DNA damage which leads to further activation of p53 and GSK3β and even more aggregation.

We also used the model to examine cellular outcomes which would be more typical of ageing, where the accumulation of DNA damage and other types of cellular damage is more gradual. As stochastic computer simulations take a long time to run, we increased the aggregation rates so that the model could be run for a reasonably short time period. Without a major damaging event, stochastic events become much more important and we see considerable variation in the time at which damage starts to accumulate. We also noted that our model predicted that tau tangles start to form before Aβ plaques but that levels of these plaques soon overtake those of tau tangles due to their more rapid increase at the elongation stage. Our results might seem contradictory to experimental data and the amyloid cascade hypothesis [34]. However, aggregates need to reach a certain size to be detectable whereas our model output can detect aggregates as soon as formation begins. If we also imposed a threshold of detection on our model output, we would see Aβ plaques before tau tangles in some simulations, depending on the threshold chosen. Most importantly, our model suggests that the formation of plaques and tangles are independent events, but that they share a common cause, namely GSK3β overactivity.

The output from our model showed that even when p53 levels rose after DNA damage, they were prevented from rising indefinitely by the action of the negative feedback loop involving Mdm2. However, due to random fluctuations in protein levels, it is possible that p53 levels would sometimes rise to levels which would exceed the threshold required to activate apoptotic pathways. In this case neuronal death would take place. Our model predicted that this was much more likely to occur when protein aggregates start to accumulate and the proteasome becomes inhibited due to less efficient turnover of p53. Interestingly studies have shown that AD patients have a lower incidence of several types of cancer [35]. So although high levels of p53 are detrimental in the ageing brain due to neuronal loss, an increase in apoptosis prevents cancer.

We used the model to test the effects of deleting either p53 or GSK3β to examine whether the interaction between GSK3β and p53 was necessary to obtain aggregation to Aβ and tau. The model shows that GSK3β is required for aggregation to take place when p53 is present but that it was possible for aggregates to form in the absence of p53, providing that GSK3β was highly expressed. A number of experiments are planned to test the model predictions. If the vicious cycle hypothesis is correct, the self-amplifying cycle of GSK3β/p53 activation should be critically dependent on both entities and should not occur when one or both are inactivated or deficient. It should be possible to monitor the status of protein aggregates, tau phosphorylation, and proteasome inhibition in cultured cells irradiated to induce DNA damage either in the presence of functional GSK3β and p53 or in conditions where a component is inhibited (for example by the GSK3β inhibitor alsterpaullone) or missing (as in cells derived from p53 null mice). The predicted role of accumulating DNA damage in triggering the GSK3β/p53 vicious cycle can be tested in cells that are genetically deficient in DNA repair components or in normal cells chronically exposed to DNA damaging agents. It will also be informative to carry out experiments with GSK3β and p53 over-expression together to see if there is enhanced Aβ production or accumulation of tau. The outcome of these experiments will be reported in a future publication.

This model only examines a very small part of the cellular system. In reality there are many pathways and many other components that we have not considered here. For example, the chaperone system is very important in maintaining protein homeostasis by binding to misfolded protein and assisting in either refolding or elimination of the damaged protein. Mitochondria also play an important role as they produce the ATP required for many cellular processes such as protein degradation. They also produce ROS as a by-product of respiration. Mitochondria, themselves are susceptible to oxidative damage and this can lead to increased levels of ROS. Other models are being developed where the emphasis is on using a systems biology approach.

When we simulated the effects of an increased aggregation rate without any initial DNA damaging event, we in effect speeded up the ageing process. Care is needed in interpreting these results as they could give the false impression that aggregation might occur at an early age. However, in reality, it is unlikely that protein homeostasis would be disturbed until old age, which is why only people over a certain age get Alzheimer's disease. However, there is considerable variation in the actual age of disease onset which may be mainly due to stochastic effects as our model suggests.

The model was kept as simple as possible but included enough detail to make some meaningful predictions. We only included degradation of unphosphorylated tau by the 20S proteasome in a ubiquitin-independent manner. Although phosphorylated tau is resistant to degradation by proteases [36], it has been shown that phosphorylated tau is degraded by ubiquitin-dependent proteasome pathway and the heat shock proteins Hsp70, Hsp70, the ubiquitin ligase CHIP and the kinase Akt are all involved [37-39]. More detailed models of Aβ and tau turnover which also contain more detail of the aggregation process are being developed and these could be easily incorporated in the current GSK3β/p53 model. We did not include turnover of GSK3β since GSK3β is relatively stable but over long time periods it could start to accumulate if the proteasome became inhibited and so it may be desirable to add detail of GSK3β turnover. Interestingly Hsp90 is also involved in maintaining the stability of GSK3β [30]. Furthermore, there are many other pathways leading to activation of GSK3β, such as deregulation of the insulin and Wnt pathways. Our current model could be used to examine cellular outcomes by assuming that if p53 reached above a certain threshold, apoptosis would take place. Alternatively we could further extend the model to include pathways leading to cell death. This would be a very useful addition to the model as neuronal loss is a common feature of neurodegenerative disorders but it occurs only in subsets of neurons in specific regions of the brain.

## Conclusions

Our model predicts that GSK3β overactivity leads to an increase in levels of Aβ plaques and tau tangles by independent processes suggesting that the observed correlation between plaques and tangles may not be due to a causal relationship. The interaction of GSK3β with p53 leads to increase activity of both proteins. Since p53 and GSK3β are both involved in the apoptotic pathway, and GSK3β overactivity leads to increased levels of plaques and tangles, our model might explain the link between protein aggregation and neuronal loss in neurodegeneration. Therefore modulating the interaction of GSK3β and p53 may be a useful therapeutic strategy.

## Competing interests

The authors declare that they have no competing interests.

## Authors' contributions

CJP built the model, ran simulations, plotted results and drafted the manuscript. DAG advised on model construction, and helped to draft the manuscript. All authors read and approved the final manuscript.

## Supplementary Material

Additional file 1 **SBML code for GSK3-p53 model with IR event**. This file contains the SBML code for the model with the event for IR included. Click here for file
